# Prophylactic para-aortic lymph node dissection in Colo-rectal cancer; pilot study

**DOI:** 10.1186/s12957-024-03515-1

**Published:** 2024-09-19

**Authors:** Abdalwahab R. Abdalwahab, Mohamed A. Abdelhamed, Mai Gad, Rasha Mahmood Allam, Alaadin Hussien

**Affiliations:** 1 Department of Surgical Oncology, National Cancer Institute, Cairo University, Cairo, Egypt; 2 Department of Surgical Pathology, National Cancer Institute, Cairo University, Cairo, Egypt; 3 Department of Epidemiology and Biostatistics, National Cancer Institute, Cairo University, Cairo, Egypt

**Keywords:** Colorectal cancer, Prophylactic, Lymph node

## Abstract

**Background:**

Colorectal cancer is the 3rd most common cancer worldwide, representing 10% of all cancer types, and is considered the 2nd leading cause of cancer-related deaths. It usually metastasizes to the liver or lung. Para-aortic lymph node metastasis is considered a metastatic disease (stage 4) according to the AJCC and is considered a regional disease (stage 3) according to the JSCCR. Para-aortic lymph node metastases occur in about 1% of cases. Neoadjuvant CTH, followed by PALN, is the best option for metastatic para-aortic LNs in colorectal cancer patients. This study addresses the value of prophylactic para-aortic LN dissection among colon-rectal cancer patients (overtreatment protocol).

**Methodology:**

This is a prospective study that included patients attending NCI, Cairo University, from December 2020 to December 2023 who were complaining of left colonic cancer or recto-sigmoid cancer and underwent left hemicolectomy, sigmoid colectomy, or LAR. All patients underwent formal mesenteric LN dissection and prophylactic para-aortic LN dissection.

**Results:**

Among 60 patients who underwent colorectal surgery with prophylactic para-aortic LN dissection, 21 cases (35%) were in the descending colon, 22 cases (36.7%) were in the sigmoid colon, 11 cases (18.3%) were in the recto-sigmoid, and 6 cases (10%) were in the upper rectum. 55 cases (91.7%) were in grade 2, and 5 cases (8.3%) were in grade 3. Neoadjuvant CTH was given in 3 cases (5%) while neoadjuvant RTH was given in 6 cases (10%). Regarding reported postoperative complications, lymphorrhea was reported in 2 patients (3.3%) and wound infection occurred in 6 patients (10%). A recurrence was reported among 8 cases (13.4%).

**Conclusions:**

We aimed in this study to highlight the value of prophylactic para-aortic lymph node dissection among colorectal cancer patients (over-treatment protocol) and report its reflection on predicting the behavior of the disease and subsequently selecting the patients who will be suitable to do this procedure.

## Introduction

Among Colo-rectal cancer patients, para-aortic lymph node metastases occur in about 1% of cases [1].

Generally, metastatic colorectal cancer management was based on systemic CTH to be followed by R0 surgical resection with an accepted curative rate, and this is now the gold standard for liver and lung metastasis [2–4].

Regarding the staging system, the American Joint Committee on Cancer (AJCC) considered PALN metastasis a metastatic disease. It categorized it as a stage IV disease, while the Japanese Society for Cancer of the Colon and Rectum (JSCCR) considered this a loco-regional disease and categorized it as a stage III disease [5–7].

The best choice for assessing the para-aortic lymph node status is the imaging data, the presence of LN size beyond 10 mm with effaced hila in CT with IV contrast is considered highly suspected for the presence of a pathological lymph node [8].

Regarding PALN metastasis, synchronous R0 resection of pathological para-aortic LNs with the primary lesion is still controversial due to a lack of prospective studies regarding overall survival and disease-free survival regarding this issue [9].

The main aim of our study is that it is a prospective study addressing patients with Colo-rectal cancer, and we did prophylactic PALN dissection for radiologically negative PALN to predict the prognosis of the disease based on the positivity of those dissected PALN and its correlations with the presence of other liver or lung metastases and its correlations with the levels of serum tumor markers, and we assess the prognosis based on our results regarding overall survival and disease-free survival.

## Methodology

### Study design and study setting

This is a prospective study—of patients attending NCI, Cairo University, from December 2020 to December 2023. We aimed to establish a strategy for prophylactic PALND. The description of predictors for pathological para-aortic lymph node metastasis, which was not evident in pre-operative radiological investigations, allows us to detect colorectal cancer patients who will benefit from the over-treatment protocol (prophylactic para-aortic lymph node dissection).

Patients were complaining of left colonic cancer or recto-sigmoid cancer and underwent either left hemicolectomy, sigmoid colectomy, or LAR; all cases were treated by one team of two colorectal surgeons. All patients underwent formal mesenteric LN dissection and prophylactic para-aortic LN dissection.

### The characteristics of participants

#### Inclusion criteria

Patients were complaining of left colonic or rectosigmoid adenocarcinoma at any stage and were candidates for surgery; radiologically, they had no suspected para-aortic pathological lymphadenopathy.

#### Exclusion criteria

Patients with radiologically suspected para-aortic pathological lymphadenopathy (more than 7 mm radiologically seen by 2 different radio-diagnosis consultants) and patients with recurrent cancer colon.

### Intervention

#### Pre-intervention evaluation

A detailed history was obtained from all participants regarding their age, sex, medical history, family history, previous malignancy, pre-operative investigations including (CEA & CA19.9), location of the tumor, pre-operative staging (based on radiological findings), pathological details, neo-adjuvant therapy, operative details including (operative procedure, operative time, intra-operative blood loss), postoperative complications including (hemorrhage, lymphorrhea & urine retention), hospital stay, final pathological details after surgery, adjuvant therapy and also we reported the recurrence during follow up which was based on serum TMs every 3 months, radiological assessment (Tri-phasic CT plus or minus MRI pelvis) every 6 months and colonoscopy every one year were also recorded.

#### Technical intervention

Under general anesthesia in the supine position, abdominal exploration was carried out through a midline incision. Mobilization of the sigmoid colon, descending colon and splenic flexure via sharp dissection of the Toldt's line, identification of the left ureter, identification of the right ureter, identification and preservation of the inferior hypogastric plexus, identification with division and ligation of the inferior mesenteric artery and vein from their origins at the level of D.J junction with orientation of the pedicle stump, identification of the right ureter, dissection of the sigmoid colon and rectum inferiorly from pre-sacral fascia in avascular plane with preservation of the inferior hypogastric plexus, dissection of the rectum anteriorly from the bladder trigon and from seminal vesicles, dissection of the rectum laterally from pelvic wall under complete vision of both ureters and both Pelvic splanchnic nerves (nervi erigentes), the rectal lesion was in mid-rectum and extending downwards till low rectum, after obtaining an adequate margins proximally, division of the colon above level of sigmoid colon and orienting the specimen proximally via silk sutures, after obtaining an adequate margins distally, division of the rectum just at the ano-rectal junction via the contour stapler and extraction of the specimen of the LAR. Performing colo-anal anastomosis via circular stapler Fr31 (Covidien) after insertion of the anvil proximally in the colon, testing anastomosis and it was watertight and airtight.

#### Technical notes

After resection of the primary tumor and performing formal mesenteric LN dissection with high ligation of the vessels from their origin in the aorta, para-aortic LN dissection was performed with landmarks of the left renal vein superiorly and bifurcation of the left common iliac artery inferiorly, with dissection of all connective tissues around the aorta and IVC Fig. 3.

### Statistical analysis

#### Statistical method

Version 26 of the SPSS (statistical package for social science) statistical tool will be used for data analysis. The mean, standard deviation, or median and range will be used to summarize numerical data. Depending on the type of data, mean ± SD was used to represent quantitative data, while numbers and percentages were used to represent qualitative data. Statistical significance was set at *p* < 0.05.

## Results

From December 2020 to December 2023, among 60 patients who underwent colorectal surgery at NCI, Cairo University, with prophylactic para-aortic LN dissection, 25 patients (41.7%) were female and 35 patients (58.3%) were male, regarding BMI. 43 patients (71.1%) were obese, 14 patients (23.3%) had medical comorbidities, and 11 patients (18.3%) had a positive family history of malignancy. Among those 60 cases reported, all patients had complaints; 21 patients (35%) had constipation, and 39 patients (65%) had constipation & bleeding per rectum. Regarding the location of the tumor, 21 cases (35%) were in the descending colon, 22 cases (36.7%) were in the sigmoid colon, 11 cases (18.3%) were in the recto-sigmoid, and 6 cases (10%) were in the upper rectum. Regarding preoperative work 46 cases (76.7%) had a low hemoglobin level below 10 gm/dl, 8 cases (13.3%) had a CEA level above 20 ng/dl, and 5 cases (8.3%) had a CA19.9 level above 35 ng/dl, 20 patients (33.3%) did triphasic CT, and 40 patients (66.7%) did triphasic CT & MRI pelvis, Among all cases, the preoperative pathological diagnosis was adenocarcinoma, 55 cases (91.7%) were in grade 2, and 5 cases (8.3%) were in grade 3. Neoadjuvant CTH was given in 3 cases (5%) while neoadjuvant RTH was given in 6 cases (10%). Regarding the operative procedure, 21 patients (35%) underwent left hemicolectomy, 16 patients (26.7%) underwent LAR, 20 patients (33.3%) underwent sigmoid colectomy, 2 patients (3.3%) underwent sigmoid colectomy with hepatic metastasectomy, and one patient (1.7%) underwent LAR with hepatic metastasectomy. Among all of our patients, the resection was in R0 fashion. Operative time was ranging from (100: 180 min.,) with a median value of 120 min., while the hospital stay was ranging from (5:17 days) with a median value of 7 days. Regarding reported postoperative complications, lymphorrhea was reported in 2 patients (3.3%) and wound infection occurred in 6 patients (10%).

We didn’t report hemorrhage, urine retention, or anastomotic leak among our cases as a postoperative complication. Regarding the pathological details of the resected specimens, the pathological variant was adenocarcinoma in all of our cases; 55 cases (91.7%) were in grade 2, and 5 cases (8.3%) were in grade 3, Harvested mesenteric LNs ranged from (17:35 LNs) with a median value of 25 among them; mesenteric LNs were positive in 36 patients (60%), while harvested para-aortic LNs ranged from (6:14 LNs) with a median value of 8 among them; PALN was positive in 10 patients (16.6%). Recurrence was reported in 8 cases (13.4%). Among those cases, 4 patients developed nodal recurrence within an interval period of more than one year and were treated via CTH followed by surgery with a good prognosis, while the other 4 cases had synchronous nodal, peritoneal, and liver metastases within an interval period of less than one year and were treated via CTH and palliative therapy with a poor prognosis Table 1.

**Table 1:** Baseline characteristics of the study population

Characteristic	Number (%)
**Sex**	
• Male	35 (58.3%)
• Female	25 (41.7%)
**BMI**	
• Obese	43 (71.7%)
• Normal range	17 (28.3%)
**Medical comorbidity**	
• Yes	14 (23.3%)
- DM	3 (21.4%)
- DM & HTN	5 (35.7%)
- HTN	6 (42.9%)
• No	46 (46.7%)
**Family history of malignancy (colorectal cancer)**	
• Yes	11 (18.3%)
• No	49 (81.7%)
**Complaint**	
• Constipation & rectal bleeding	39 (65%)
• Constipation only	21 (35%)
**Location of the tumor**	
• Descending colon	21 (35%)
• Sigmoid	22 (36.7%)
• Recto-sigmoid	11 (18.3%)
• Upper rectum	6 (10%)
**Laboratory investigations**	
• HB level (gm/dl)	
- More than 10	46 (76.7%)
- Less than 10	14 (23.3%)
• CA19.9 level (ng/dl)	
- More than 35	5 (8.3%)
- Less than 35	55 (91.7%)
• CEA level (ng/dl)	
- More than 20	8 (13.3%)
- Less than 20	52 (86.7%)
**Radiological investigations**	
• MRI pelvis & triphasic CT	40 (66.7%)
• Triphasic CT only	20 (33.3%)
**Pathological data (pre-operative)**	
• Pathological variant of tumor	
- Adenocarcinoma	60 (100%)
• Pathological grade	
- Grade 2	55 (91.7%)
- Grade 3	5 (8.3%)
**Neo-adjuvant therapy**	
• Neoadjuvant chemotherapy	
- Yes	3 (5%)
- No	57 (95%)
• Neoadjuvant radiotherapy	
- Yes	6 (10%)
- No	54 (90%)
**Operative details**	
• Operative procedure	
- Left hemicolectomy	21 (35%)
- Sigmoid colectomy	20 (33.3%)
- Sigmoid colectomy & hepatic metastasectomy	2 (3.3%)
- LAR	16 (26.7%)
- LAR & hepatic metastasectomy	1 (1.7%)
• Operative time (in minutes)	
- Less than 120 min	42 (70%)
- More than 120 min	18 (30%)
**Hospital stay (in days)**	
• Less than 7 days	43 (71.7%)
• More than 7 days	17 (28.3%)
**Post-Operative Complications**	
• Wound infection	
- Yes	6 (10%)
- No	54 (90%)
• Lymphorrhea	
- Yes	2 (3.3%)
- No	58 (96.7%)
**Pathological data (post-Operative)**	
• Pathological variant	
- Adenocarcinoma	60 (100%)
• Grade	
- Grade 2	55 (91.7%)
- Grade 3	5 (8.3%)
• Depth of invasion	
- Infiltrating non-periodized fat	60 (100%)
• Margins of resection	
- Negative margins (R0 resection)	60 (100%)
- Positive margins (R1 or R2 resection)	Zero
• Lymphovascular invasion	
- Positive	36 (60%)
- Negative	24 (40%)
• Perineural invasion	
- Positive	10 (16.7%)
- Negative	50 (83.3%)
• Resected Mesenteric LNs	
- Number harvested	
# More than 25 LNs	23 (38.3%)
# Less than 25 LNs	37 (61.7%)
- Positive LNs	36 (60%)
- Negative LNs	24 (40%)
• Resected Para-aortic LNs	
- Number harvested	
# More than 8 LNs	23 (38.3%)
# Less than 8 LNs	37 (61.7%)
- Positive LNs	10 (16.6%)
- Negative LNs	50 (83.4%)
• Resected HFLs among hepatic metastasectomy cases	3 (100% among those cases)
**Recurrence**	
• Site of recurrence	
- Nodal recurrence	4 (6.7%)
- Multiple recurrence sites (nodal, peritoneal & liver)	4 (6.7%)
• Interval time	
- Less than one year	4 (6.7%)
- More than one year	4 (6.7%)
• Treatment	
- CTH then surgery	4 (6.7%)
- CTH and palliative therapy	4 (6.7%)
• Fate & prognosis	
- Good prognosis	4 (6.7%)
- Poor prognosis	4 (6.7%)

Data presented as numbers and percentages as appropriate

*P* values are determined by the Chi-square test (χ2) & the Student t-test

*Abbreviations*: *DM* Diabetes mellitus, *HTN* Hypertension, *BMI* Body Mass Index

**Fig. 1:**
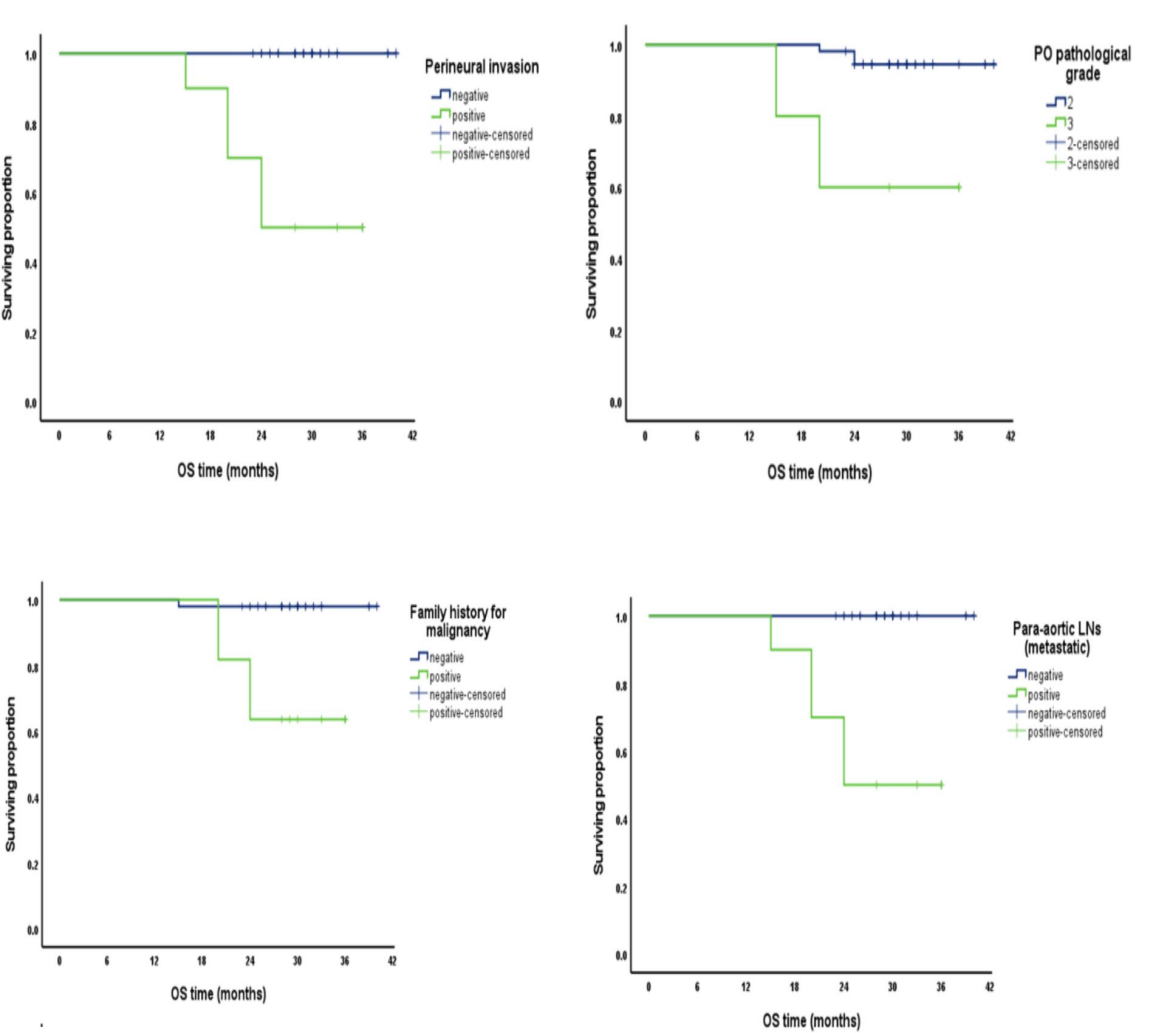
Factors related to Overall survival (OS)

We reported that factors associated with positive PALN include: positive family history of malignancy (*p*-value < 0.001), CEA (ng/dl) > 20 (*p*-value < 0.001), CA19-9 (ng/dl) > 35 (*p*-value < 0.001), pathological grade 3 (*p*-value < 0.001), M1 stage (*p*-value = 0.004), positive lymph vascular invasion (*p*-value = 0.005), positive perineural invasion (*p*-value < 0.001), and positive mesenteric LNs (metastatic) (*p*-value = 0.005). Regarding survival analysis, regarding overall survival (OS) on univariate analysis, cumulative survival proportion at 3 years was reported to be with a negative family history for malignancy with a significant *p*-value (> 0.001), with a CEA level < 20 ng/dl with a *p*-value (0.04), with a CA19.9 level > 35 ng/dl with a *p*-value (0.002), with a pathological grade 2 with a *p*-value (0.002), with a negative PALN with a significant *p*-value (> 0.001), and with a negative perineural invasion with a significant *p*-value (> 0.001). A multivariate analysis of OS reported that pathological grade was related to cumulative OS with a *p*-value (0.013) Fig. 1.

**Fig. 2:**
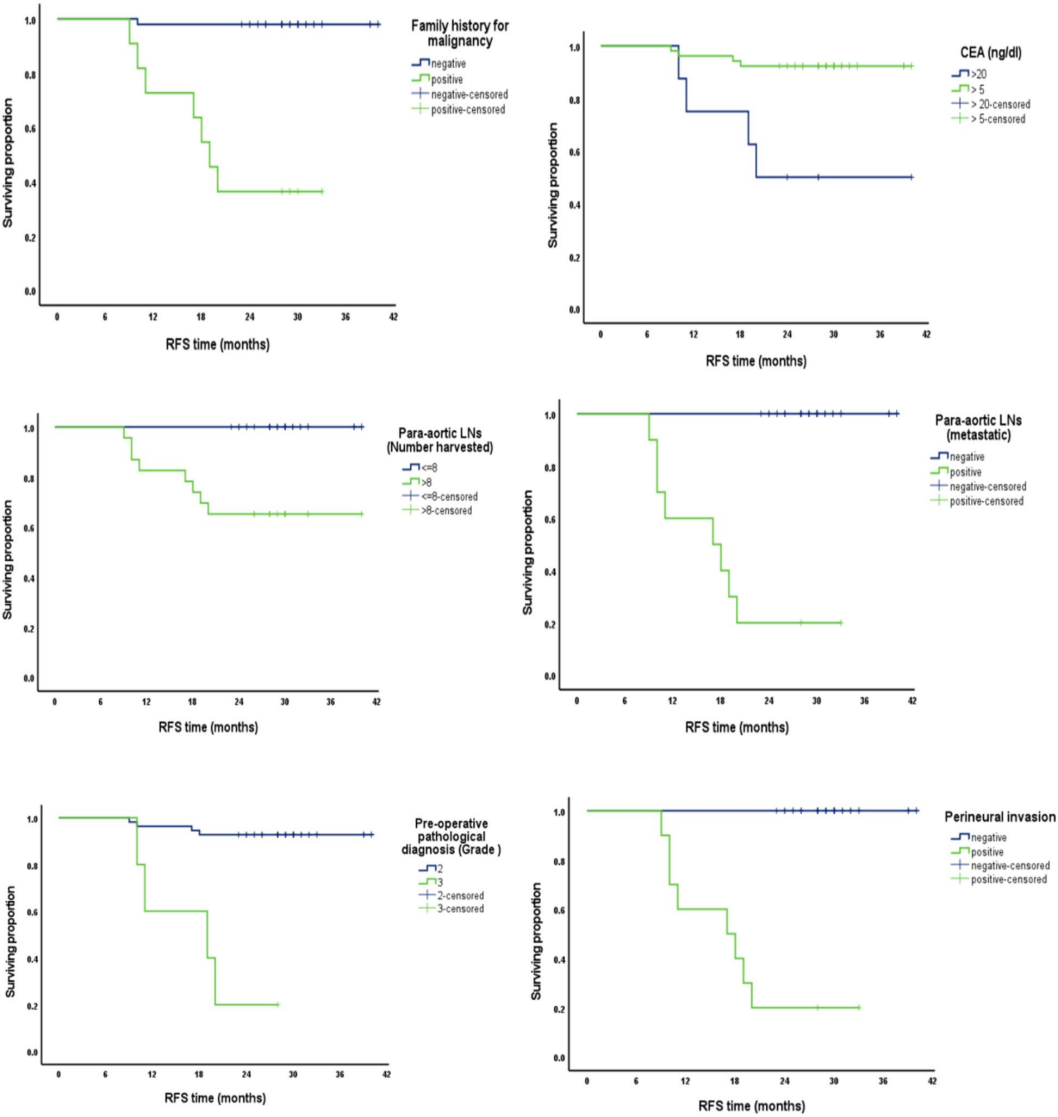
Factors related to Recurrence free survival (RFS)

Regarding recurrence-free survival (RFS), on univariate analysis, cumulative survival proportion at 3 years was reported to be with a negative family history of malignancy, CEA level > 20 ng/dl, grade 2, negative PALN, and negative perineural invasion with a significant *p*-value (> 0.001). A multivariate analysis of RFS reported that pathological grade was related to cumulative RFS with a significant *p*-value (0.001) Fig. 2.

## Discussion

Para-aortic lymph node dissection in Colorectal cancer patients was first reported by Dr. Deddish in 1950 [10].

Reported complications from para-aortic lymph node dissection include urinary dysfunction and sexual dysfunction. Leggeri et al. reported that there was no reflected improvement in recurrence rate or overall survival in Colorectal cancer patients who underwent prophylactic para-aortic lymph node dissection [11].

**Fig. 3:**
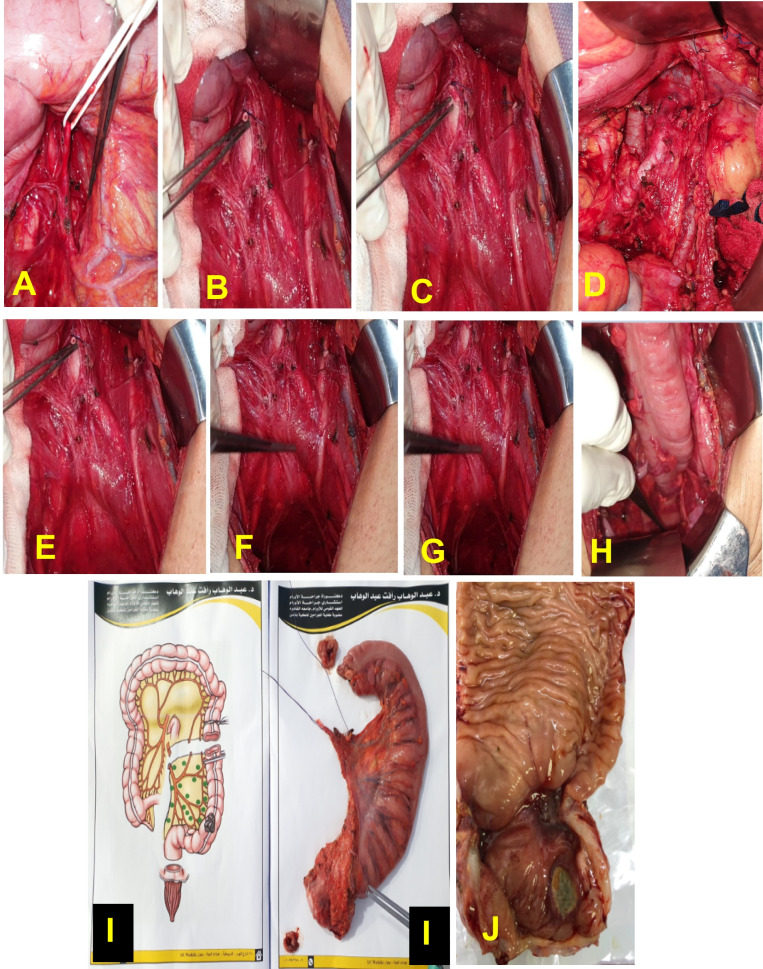
Steps for LAR with PALND: (**A**) suspension of the inferior mesenteric artery, (**B** and **C**) stump of the inferior mesenteric artery after division and ligation at its origin from aorta, (**D**) para-aortic LN dissection, (**E**, **F** and **G**) preservation of the inferior hypogastric plexus and both pelvic splanchnic nerves, (**H**) Colo-anal anastomosis, (**I**) extracted specimen, (**J**) division of the specimen by the pathologist

Among our series, we reported a complication rate in 8 patients (13.3%); lymphorrhea was reported in 2 patients (3.3%); and wound infection occurred in 6 patients (10%). We didn’t report any cases complaining of sexual dysfunction among male patients based on the International Index of Erectile Function as we were kept on preserving neurological erectors Fig. 3.

Many of the updated studies about PALN in Colorectal cancer reported improved survival after therapeutic PALN for metastatic para-aortic LNs with post-operative morbidity (7.8: 38.9%), but most of those studies were retrospective [12–16].

Ogura et al. proposed a model to predict suspected pathological para-aortic lymph nodes in Colorectal cancer patients to perform therapeutic PALND. This model involves the status of lymphovascular invasion, mesenteric nodal status, elevated CEA level, and radiologically visible para-aortic lymph node greater than 10 mm [17].

Choi et al., among 24 Colo-rectal cancer cases, reported that the presence of two suspected pathologically para-aortic lymph node metastases is enough to decide to perform therapeutic PALND [18].

## Conclusion

Our study demonstrates the value of prophylactic PALND in left colonic, sigmoid, and recto-sigmoid adenocarcinomas and its reflection on the prediction of the behavior and prognosis of the disease. This study is considered one of a few to continue focusing on the issue of prophylactic PALND in the future, but with increasing sample size and making the study multicenter. That prescribes the value of prophylactic PALND. We aimed to continue focusing on the issue of prophylactic PALND in the future but with increasing sample size and making the study multicenter.

## Data Availability

The corresponding author can provide the dataset and materials used in this pilot project upon reasonable request. The dataset contains follow-up data, clinical characteristics, operation details, pathology findings, and anonymized patient demographic data. Standardized protocols were followed in the collection and management of all data to guarantee patient privacy. The publication includes a detailed description of the data collection methodologies, surgical techniques, and clinical protocols. The authors have provided the statistical code that was utilized for the data analysis. The authors are happy to share the dataset with reputable researchers who agree to suitable data use and privacy standards, but the whole dataset cannot be made publicly available due to privacy limitations.

## Abbreviations

PALN: Para-aortic LN
PALND: Para-aortic LN dissection
RTH: Radiotherapy
AJCC: The American Joint Committee on Cancer
JSCCR: The Japanese Society for Cancer of the Colon and Rectum
CTH: Chemotherapy
LAR: Low Anterior Resection
RFS: Recurrence-free survival

## Data availability

No datasets were generated or analysed during the current study.
